# Adipocytes promote prostate cancer stem cell self-renewal through amplification of the cholecystokinin autocrine loop

**DOI:** 10.18632/oncotarget.6643

**Published:** 2015-12-17

**Authors:** Kai-Dun Tang, Ji Liu, Lidija Jovanovic, Jiyuan An, Michelle M. Hill, Ian Vela, Terence Kin-Wah Lee, Stephanie Ma, Colleen Nelson, Pamela J. Russell, Judith A. Clements, Ming-Tat Ling

**Affiliations:** ^1^ Australian Prostate Cancer Research Centre-Queensland & Institute of Health and Biomedical Innovation, Queensland University of Technology and Translational Research Institute, Woolloongabba, QLD, Australia; ^2^ The University of Queensland Diamantina Institute, The University of Queensland, Translational Research Institute, Brisbane, QLD, Australia; ^3^ Department of Pathology, Faculty of Medicine, The University of Hong Kong, Hong Kong, SAR, China; ^4^ Department of Anatomy, Faculty of Medicine, The University of Hong Kong, Hong Kong, SAR, China

**Keywords:** cholecystokinin, adipocytes, prostate tumor-initiating cells

## Abstract

Obesity has long been linked with prostate cancer progression, although the underlying mechanism is still largely unknown. Here, we report that adipocytes promote the enrichment of prostate cancer stem cells (CSCs) through a vicious cycle of autocrine amplification. In the presence of adipocytes, prostate cancer cells actively secrete the peptide hormone cholecystokinin (CCK), which not only stimulates prostate CSC self-renewal, but also induces cathepsin B (CTSB) production of the adipocytes. In return, CTSB facilitates further CCK secretion by the cancer cells. More importantly, inactivation of CCK receptor not only suppresses CTSB secretion by the adipocytes, but also synergizes the inhibitory effect of CTSB inhibitor on adipocyte-promoted prostate CSC self-renewal. In summary, we have uncovered a novel mechanism underlying the mutual interplay between adipocytes and prostate CSCs, which may help explaining the role of adipocytes in prostate cancer progression and provide opportunities for effective intervention.

## INTRODUCTION

Prostate cancer is the second most common cause of solid tumours in men worldwide [1]. Despite recent advances in the detection of early prostate cancer, there remains no effective therapy for patients with metastatic disease [2]. The majority of patients with advanced disease will respond initially to androgen ablation therapy. However, 75-80% of them will go on to develop bone metastasis and once the tumor established in the bone, the disease is considered as incurable [3-5].

Tumor metastasis develops when cancer cells disseminate into the circulation, colonize secondary tissues and redevelop into bulk tumors [6]. Recent evidence supports the idea that tumor metastasis originates from a rare population of cancer cells known as cancer stem cells (CSCs). CSCs are characterized by their highly invasive characteristics and by their ability to self-renew and differentiate into heterogeneous lineages of cancer cells [7]. The unique plasticity of CSCs also allows them to undergo the phenotypic switch known as the epithelial-to-mesenchymal transition (EMT) [8], which facilitates the mobilization and homing of tumor cells to target organs [9]. The stemness of CSCs is highly dependent on the presence of a stem cell niche. Recent studies suggested that CSCs are capable of creating their own niche by recruiting mesenchymal stem cells or macrophages, resulting in expansion of the CSC population within the tumor microenvironment [10, 11]. The same process is suggested to occur during the development of bone metastasis, whereby disseminated prostate and breast tumor cells with CSC properties have been found to occupy the hematopoietic stem cell niche and hijacking the signalling pathways within bone marrow [12-14]. Therefore, identifying the key components of the CSC niche that support prostate cancer metastasis may offer opportunities for new treatment strategies.

Emerging data from recent studies support that adipocytes play a key role in prostate tumor metastasis. For example, obesity, which is associated with abnormal growth and functions of adipocytes, has been shown to correlate strongly with tumor metastasis in prostate cancer patients. Meanwhile, high-fat diet has also been consistently shown to promote the development of prostate tumor metastasis [15]. Furthermore, adipocytes isolated from periprostatic adipose tissues were found to induce invasiveness of prostate cancer cells [16]. Recently, adipocytes have also been reported to stimulate the growth and aggressiveness of prostate cancer cell through the production of a number of adipokines [17-20]. Considering that adipocyte lineage cells were found to stimulate follicular stem cell expansion [21], it is possible that adipocytes may promote prostate tumor metastasis by contributing to the formation of a CSC niche within the tumor microenvironment.

Here, we demonstrated the role of adipocytes in supporting self-renewal of prostate CSCs. We found that co-culturing of prostate cancer cells with adipocytes resulted in CSC enrichment, which was associated with upregulation of cholecystokinin (CCK), a peptide hormone regulating fat digestion and satiety. CCK not only functions as an autocrine factor to promote CSC self-renewal, but also acts as a paracrine factor on adipocyte to stimulate the secretion of the cysteine protease cathepsin B (CTSB). Surprisingly, CCK secretion by the cancer cells was found to be induced by CTSB, suggesting that CCK and CTSB contribute to an autocrine/paracrine amplification loop that mediates the mutual interplay between prostate CSCs and adipocytes.

## RESULTS

### Adipocytes promote prostate CSC self-renewal

**Figure 1:**
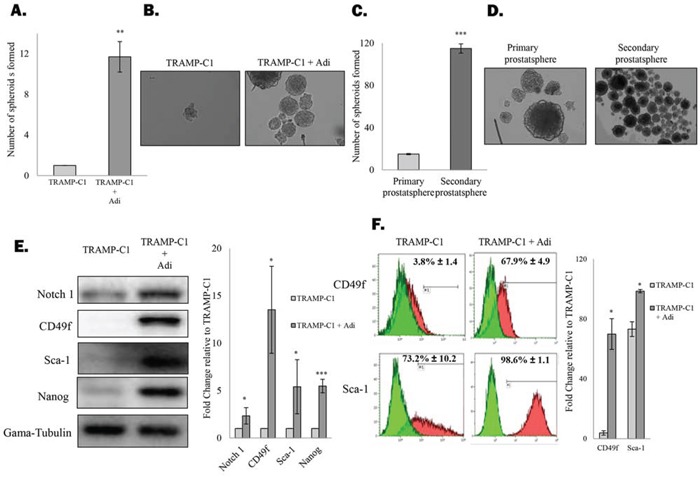
Adipocytes promote prostate CSC self-renewal **A.** Adipocytes stimulate formation of prostaspheres. TRAMP-C1 cells were seeded in ultra-low attachment plates in the presence or absence of 3T3-L1-derived adipocytes. After 7 days, prostaspheres formed were counted under the microscope. **B.** Representative images of the prostaspheres. **C&D.** Primary TRAMP-C1 prostasphere were dissociated and were allowed to grow as secondary prostaspheres in the presence of adipocytes. **E&F.** Expression of CSC markers by TRAMP-C1 cells or the prostatsphere formed under the co-culture conditions were examined by western blotting (Notch 1, CD49f, Sca-1 and Nanog) and flow cytometry (CD49f and Sca-1) respectively. The results are presented as the mean ± SD from triplicate experiments. (p values: * < 0.05, ** < 0.005, *** < 0.0005).

In order to understand the mutual interplay between adipocytes and prostate CSCs population, mouse prostate cancer cell line (TRAMP-C1) was allowed to grow in a non-adherent condition in the presence or absence of adipocytes (derived from the mouse 3T3-L1 pre-adipocyte cell line). Co-culturing with fully differentiated adipocytes strongly induced the self-renewal ability of the TRAMP-C1 cells, as evidenced by the drastic induction of spheroid formation (1 vs 12) under co-culture conditions (Figure 1A&1B). Apart from increasing the number of prostaspheres formed, the size of individual prostaspheres was also significantly increased in the presence of adipocytes adipocytes (Figure 1B). Similarly, adipocytes also promoted spheroid formation of three other mouse prostate cancer cell lines TC1-T5, RM1 and RM1-BM (Suppl. Figure 1). Meanwhile, adipocytes also significantly induced the formation of secondary prostaspheres (Figure 1C&1D), further confirming the effect of adipocytes on prostate CSC self-renewal ability. Consistently, mouse bone marrow derived adipocytes (OP9) and human SGBS adipocytes also promoted the prostatsphere formation of TRAMP-C1 and PC-3 cells to a similar extent respectively (Suppl. Figure 2 and data not shown), suggesting that the CSC-promoting effect may be common among adipocytes derived from different origins.

To validate our findings, western blotting and flow cytometry analysis of common CSC markers were performed. As shown in Figure 1E, protein expression of Notch1, CD49f, Sca-1 and Nanog in TRAMP-C1 cells was significantly upregulated after co-culturing with adipocytes. Furthermore, the percentage of cells expressing the two putative CSC markers CD49f and Sca-1 was significantly upregulated after co-culturing with adipocytes (Figure 1F). These results confirm that adipocytes actively promote self-renewal of prostate CSCs, leading to subsequent expansion of CSC population, supporting the notion that adipose tissues may contribute to a metastatic niche necessary for the maintenance of prostate CSCs.

### Adipocytes induce a CCK autocrine loop in prostate CSCs

**Figure 2:**
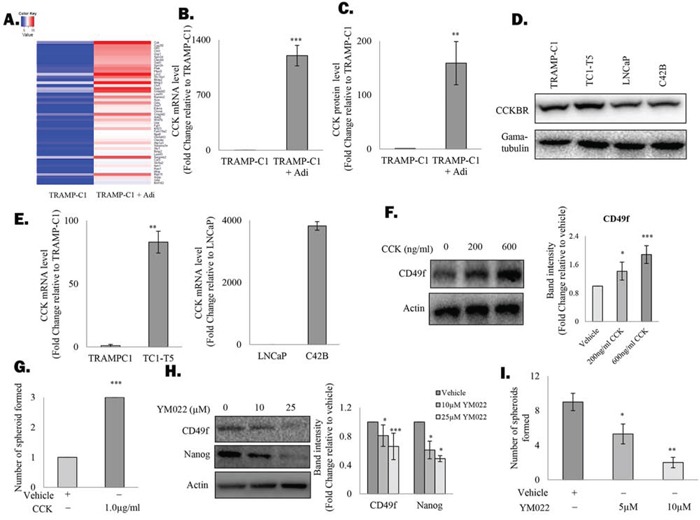
Adipocytes induce the cholecystokinin autocrine loop in prostate CSCs **A.** Genes upregulated in TRAMP-C1 prostatsphere after co-culturing with adipocytes (grown in an insert) were identified with cDNA microarray analysis and the top 50 genes were displayed as a heat map. **B.** Upregulation of CCK mRNA in TRAMP-C1 prostaspheres was validated with RT-PCR. **C.** ELISA was performed to confirm the induction of CCK production in TRAMP-C1 cells after co-culturing with adipocytes. **D.** CCKBR expression was analysed in mouse (TRAMP-C1, TC1-T5) and human prostate cancer cell lines (LNCaP and C42B) using western blotting. **E.** CCK is upregulated in metastatic prostate cancer cells. mRNA level of CCK in TRAMP-C1, LNCaP and their corresponding metastatic sublines (TC1-T5 and C42B) were analysed by RT-PCR. **F.** Effect of CCK treatment on CSC marker expression in prostate cancer cells. TRAMP-C1 cells were treated with recombinant CCK for 48 hours and the expression of CD49f was examined using western blotting. **G.** CCK promotes prostasphere formation by TRAMP-C1 cells. TRAMP-C1 cells were seeded in an ultra-low attachment plate in the presence or absence of human recombinant CCK. After 7 days, prostaspheres formed were counted under the microscope. **H.** Inhibition of CCKBR downregulated CSC marker expression (CD49f) in TRAMP-C1 cells. Cells treated with either the vehicle or CCKBR inhibitor (YM022 ) (10 and 25 μM) were lysed and subjected to western blot analysis against the indicated proteins. **I.** Inhibition of CCKBR abolishes the effect of adipocytes on prostasphere formation. TRAMP-C1 cells co-cultured with adipocytes were treated with different doses of the YM022 (5 and 10 μM) and the prostaspheres formed under each condition were counted under microscope. All experiments were repeated at least three times, and the results are presented as the mean ± SD. (p values: * < 0.05, ** < 0.005, *** < 0.0005).

To understand the underlying mechanism that drives the active self-renewal of prostate CSCs under the co-culture conditions, cDNA microarray analysis was performed to compare the gene expression profile of TRAMP-C1 cells that grow alone or in the presence of adipocytes. As expected, expression of a number of known stemness factors such as ALDH1A1, GFI-1 or ITGA2 were highly induced in TRAMP-C1 cells in the presence of adipocytes (Figure 2A and Suppl. Figure 3). However, the gene that showed the highest level of induction was found to encode the protein CCK (Figure 2A and Suppl. Table 1), a peptide hormone expresses mainly by the mucosal epithelium in response to consumption of high-fat diet. Subsequent analysis with Real time- polymerase chain reaction (RT-PCR) and Enzyme-linked immunosorbent assay (ELISA) confirmed that both CCK mRNA and protein secretion were upregulated in prostate cancer cells in the presence of adipocytes (Figure 2B&2C and Suppl. Figure 4). Surprisingly, prostate cancer cell lines were found to actively expressing the CCK receptor CCKBR (Figure 2D). Since activation of CCKBR has recently been shown to promote the stemness of colon cancer cells [22], it is possible that the induction of CCK expression by adipocytes may lead to the activation of an autocrine loop and as a result promote CSC self-renewal. Indeed, while CCK mRNA was expressed at low level in both TRAMP-C1 and LNCaP cells, the corresponding bone metastatic sublines (i.e. TC1-T5 and C42B), which are enriched with CSC population (Suppl. Figure 5), were both found to express higher level of CCK transcript (Figure 2E). Meanwhile, treatment with human recombinant CCK not only induced the expression of CSC marker in TRAMP-C1 cells (Figure 2F), but also promotes the formation of prostasphere by the cells (Figure 2G), indicating the importance of CCK in regulating prostate CSC self-renewal. To confirm our findings, TRAMP-C1 cells were treated with a CCKBR specific inhibitor (i.e. YM022). As shown in Figure 2H, inhibition of CCKBR was found to suppress CSC marker expression in TRAMP-C1 cells. More importantly, CCKBR inhibition was found to suppress the promoting effect of adipocytes on spheroid formation of the cancer cells (Figure 2I), supporting that adipocytes stimulate prostate CSC self-renewal by facilitating the activation of the CCK/CCKBR autocrine loop.

### CCK stimulates CTSB secretion of the adipocytes

**Figure 3:**
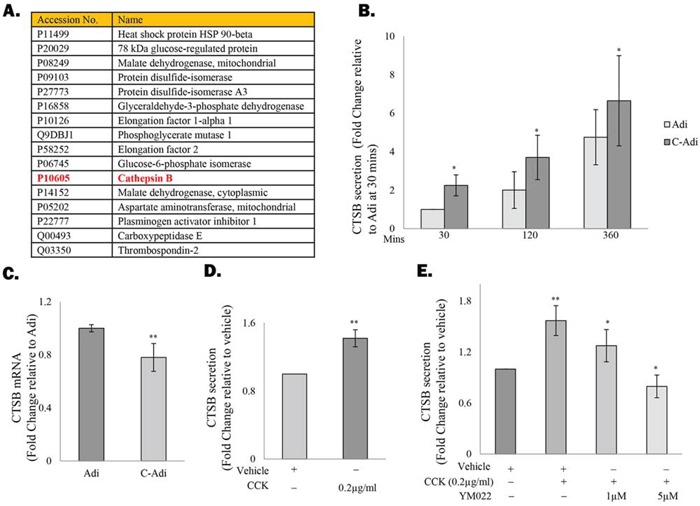
CCK stimulates CTSB secretion by the adipocytes **A.** Protein secretion profiles of 3T3-L1-derived adipocytes before or after being co-cultured with TRAMP-C1 cells was determined by mass spectrometry. Proteins upregulated under the co-culture condition were listed in Table A. **B&C.** ELISA and RT-PCR were performed to validate the induction of CTSB expression in adipocytes after co-culture with TRAMP-C1 cells. (D&E) Secretion of CTSB in adipocytes is regulated by CCK. **D.** 3T3-L1-derived adipocytes were treated with CCK recombinant protein (0.2μg/ml) for 4 hours and the level of CTSB in the conditioned medium was analysed by ELISA. **E.** Addition of YM022 (1 and 5 μM) completely abolished the effects of CCK on CTSB induction. Each experiment was repeated at least three times, and the results are presented as the mean ± SD. (p values: * < 0.05, ** < 0.005).

Since CCK has been shown to regulate the function of adipocytes through activation of the CCKBR, we therefore speculate that by actively secreting CCK, prostate CSCs may also influence the co-cultured adipocytes, possibly in a way that favour their self-renewal ability. Indeed, mass spectrometry analysis of the conditioned medium (CM) from adipocytes before and after co-cultured with prostate CSCs revealed that the cysteine protease CTSB, which plays an important role in prostate cancer progression [23], was among the proteins induced in adipocytes under the co-culture condition (Figure 3A). Subsequent analysis of the CM with ELISA further confirmed that CTSB secretion was upregulated in adipocytes that have been co-cultured with prostate CSCs (Figure 3B), although the mRNA level of CTSB was not induced under the same condition (Figure 3C). Similarly, CTSB secretion, but not the mRNA level (data not shown), was induced in adipocytes that were treated with recombinant CCK (Figure 3D). However, the effect of the recombinant CCK was abolished in the presence of YM022 (Figure 3E), confirming that CCK induced CTSB through activation of the CCKBR.

### CCK and CTSB contributes to an autocrine/paracrine amplification loop

**Figure 4:**
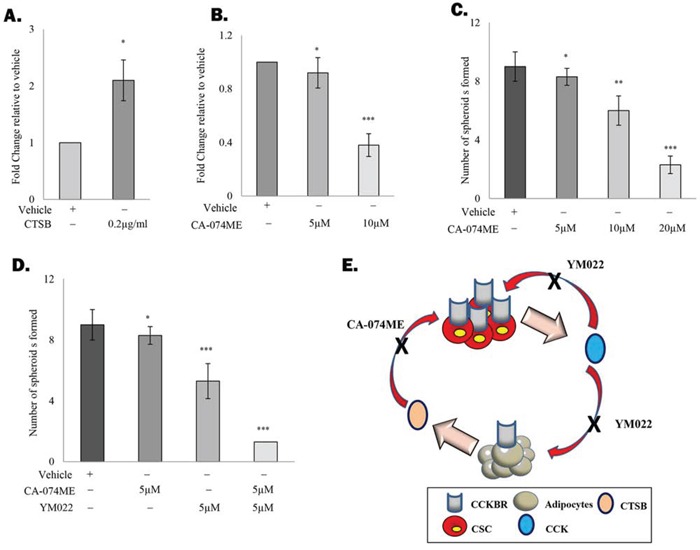
CCK and CTSB contribute to an autocrine/paracrine amplification loop **A.** CTSB regulates the secretion of CCK by prostate cancer cells. TC1-T5 cells were treated with recombinant CTSB protein (0.2μg/ml) for 4 hours and the level of CCK in the conditioned medium was determined by ELISA. **B.** Inhibition of CTSB with CTSB inhibitor CA-074ME (10μM) suppresses CCK secretion in TC1-T5 cells. **C.** CTSB inhibition suppressed the promoting effect of adipocytes on prostatsphere formation in a dose dependent manner. TRAMP-C1 cells co-cultured with 3T3-L1-derived adipocytes were treated with different dosage of CA-074ME (5, 10 and 20μM). Prostaspheres were counted and imaged at day 7. **D.** CTSB inactivation sensitizes prostate CSCs to YM022 treatment. TRAMP-C1 cells co-cultured with 3T3-L1-derived adipocytes were treated with the YM022 alone or in combination with CA-074ME. After 7 days, prostaspheres formed were counted and imaged under the microscope. **E.** Summary of the proposed role of adipocytes on prostate CSC maintenance. Each experiment was repeated at least three times, and the results are presented as the mean ± SD. (p values: * < 0.05, ** < 0.005, *** < 0.0005).

One of the key functions of cathepsin protein family is the regulation of peptide hormone release and activation [24]. For example, CSTB has been shown to promote the liberation of thorexine at the apical membrane [25]. Meanwhile, cathepsin L1 was also found to induce the processing of pro-CCK, leading to an increase in the secretion of the active CCK [26]. Although CTSB has not been reported to regulate CCK secretion, we found that treatment of TRAMP-C1 cells with recombinant CTSB resulted in significant upregulation of CCK level in the conditioned medium (Figure 4A) without affecting CCK mRNA levels (data not shown). Meanwhile, inhibition of CTSB significantly suppressed CCK production by the cancer cells, further supporting that CTSB regulates CCK secretion (Figure 4B). Since CCK is also capable of inducing CTSB production of adipocytes, we anticipated that this autocrine/paracrine amplification loop may play a key role in sustaining the adipocyte-promoted prostate CSC self-renewal. Consistent with our hypothesis, the promoting effect of adipocytes on prostasphere formation was significantly suppressed in the presence of CTSB inhibitor (CA-074ME) (Figure 4C). Meanwhile, CA-074ME was found to significantly enhance the effect of YM022 against prostasphere formation (Figure 4D). These results clearly indicate the critical role of this novel autocrine/paracrine loop in mediating the effect of adipocytes on prostate CSC self-renewal Summarized in Figure 4E.

## DISCUSSION

The frequent invasion of local tumor into periprostatic adipose tissues and the metastasis of advanced tumors into the adipocyte-rich bone marrow clearly suggest the importance of adipose tissues during prostate cancer progression [27, 28]. What is not yet clear is how prostate cancer cells interact with the adipocytes to support their eventual expansion into metastatic tumor. Here, we report for the first time that adipocytes actively interplay with prostate CSCs through a novel autocrine/paracrine amplification loop, leading to rapid enrichment of the CSC population.

Similar to normal stem cells, the stemness of prostate CSCs is highly dependent on the presence of a stem cell niche [29]. The stromal cells within the primary prostate tumor appear to play a key role in promoting the tumorigenicity of the prostate cancer cells, as evidenced by the ability of cancer associated fibroblasts in transforming normal prostate epithelial cells [30]. Similarly, our finding that adipocytes actively stimulate prostate CSC self-renewal support the proposal that adipose tissues may contribute to a metastatic niche necessary for the maintenance of prostate CSCs. Since obesity is associated with adipocyte dysfunction and abnormal adiposity, the resulting enrichment of the metastatic niches (i.e. the periprostatic adipose tissues and bone marrow fat) may in turn contribute to the frequent tumor metastasis and poor prognosis observed in obese prostate cancer patients [15].

The significant upregulation of CCK mRNA in prostate cancer cells upon co-cultured with adipocytes suggests that CCK is playing an important role in mediating the effect of adipocytes on CSC self-renewal. Expression of CCK was believed to be restricted to the enteroendocrine cells and to specialized neurons within the brain [31, 32]. It is therefore surprising that CCK and its receptor were both found to be expressed by prostate cancer cells. Our study therefore represents the first to discover the autocrine function of CCK in the maintenance of prostate CSCs. Exactly how CCK promotes the stemness of prostate CSCs is still unclear, although the binding and activation of CCKBR by gastrin, a hormone peptide structurally related to CCK, was found to regulate the proliferation of normal and malignant colon stem cells through suppression of both BMP-2 and Id4 expression [22]. Since CCK also binds to and activates CCKBR, it is possible that CCK may act through the same downstream signalling pathway.

Dietary fat is the major risk factor not only for obesity, but also for prostate cancer progression [33, 34]. Intake of dietary fat is known to induce a rapid upregulation of CCK expression by the enteroendocrine cells within the intestine [35]. Therefore, long term high-fat diet consumption is expected to result in chronic elevation of serum CCK level. Indeed, in a recent study, mice that consumed a high-fat diet were found to have ten fold higher of serum CCK level when compared to the control group [36]. In pancreatic cancer, CCK expression was also found to associate with larger tumors and a higher incidence of metastasis [36, 37]. Considering that prostate cancer cells also express CCKBR and respond to CCK stimulation, the effects of high-fat diet on prostate tumor progression reported previously [38] may well be a consequence of CCK elevation. Therefore, exposing the cancer cells to a high lipid content (i.e. high-fat diet or co-culturing with adipocytes) may promote the CCK-autocrine loop and further driven the expansion of prostate CSCs.

It is worth noting that CCK not only regulates fat and protein digestion, but also controls the level of satiety. In an animal study, systemic administration of CCK was found to induce anorexia [39]. Meanwhile, serum CCK level was found to be significantly higher in older individuals [40], and is suggested to contribute to aging-associated anorexia. Since we found that CCK expression is highly upregulated in bone metastatic prostate cancer cells, it is possible that CCK secreted by cancer cells may contribute to the anorexia commonly found in advanced stage cancer patients, although further examination of serum CCK level in prostate cancer patients is needed to confirm this hypothesis.

Our findings suggest that the secretion of CCK by prostate CSCs may also play roles in modulating the function of adipocytes, possibly in a way that further facilitates CSC expansion. One of the key pieces of evidence is the stimulation of CTSB secretion by the adipocytes, which in turn further promotes the secretion of CCK by the cancer cells. Our discovery of this seemingly paracrine/autocrine amplification loop may help in explaining the mutual interplay between prostate cancer cells and adipocytes. More importantly, despite the presence of adipocytes, disruption of this loop by means of inactivating either CCK receptor or CTSB can significantly inhibit prostate CSC expansion, clearly demonstrating the therapeutic potential of this paracrine/autocrine amplification loop for targeting CSC population.

In summary, we have identified a novel mechanism that mediates the interaction between adipocytes and prostate cancer cells, which may offer opportunities for the development of new treatment against metastatic prostate cancer.

## MATERIALS AND METHODS

### Spheroid formation assay

The spheroid formation assay was modified from a previously reported protocol [41, 42]. Briefly, cells were harvested and counted using a Scepter™ Automated Cell Counter (Millipore, Billerica, MA, USA). Four hundred cells were added to each well of a 24-well ultra-low attachment plates (Sigma-Aldrich, St. Louis, MO, USA). Adipocytes differentiated from the 3T3-L1 cells were then added into a cell culture insert (0.4 μM) (Millipore) placed inside the well. Cells were grown in DMEM/F12 (Invitrogen, Carlsbad, CA, USA), supplemented with 10 μg/mL insulin (Sigma-Aldrich), B27 (Invitrogen), 80 ng/mL EGF (Sigma-Aldrich) and 40 ng/mL basic FGF (Invitrogen). The number of spheroids formed was counted at Day 7 or at the end of the treatment. Each experiment was performed in triplicate and was repeated at least 3 times, with each data point represents the mean and SD. Statistical difference was determined by Student's t test and was considered as significant if p < 0.05.

### Western blot

Experimental procedures have been described in our previous study [43]. Firstly, cell pellets were collected and lysed with lysis buffer (Cell Signalling Technology, Danvers, MA, USA) containing 100 μM phenylmethylsulfonyl fluoride (PMSF; Sigma-Aldrich). The cell lysates were quantified using the Pierce™ BCAProtein Assay Kit (Thermo Fisher Scientific, Rockford, IL, USA) before loading onto a SDS-polyacrylamide gel. The resolved proteins were then transferred onto a PVDF membrane (Millipore) which was then blocked with 10% skim milk in TBST-T buffer at room temperature. After the blocking, the membrane was probed with the indicated antibody for 1 hour at room temperature prior to being washed with TBS-T. The membrane was then incubated with the corresponding secondary antibody for another hour at room temperature. After washing with TBS-T, the membrane was incubated with Immobilon Western Chemiluminescent HRP Substrate (Millipore), and the signals were visualized and quantified using a Bio-Rad ChemiDoc™ XRS Gel Documentation System.

### Flow cytometry analysis

Experimental procedures have been described by the manufacturer. Briefly, cells were collected, washed twice with phosphate-buffered saline (PBS) and subsequently resuspended in 50 μl of fluorescence-activated cell sorting (FACS) buffer (0.02% sodium azide and 2% FBS in PBS) before incubating with the fluorescent dye-conjugated antibodies at 4°C in the dark for 30 minutes. After incubation, the cells were washed twice with PBS and resuspended in 200 μl of FACS buffer, and the flow cytometry analysis was performed using the BD™ LSR II. The results were then analysed using the KALUZA software.

### Microarray analysis

TRAMP-C1 cells that were grown alone or co-cultured with adipocyte were lysed for RNA extraction with the RNeasy Mini Kit (Qiagen, Germantown, MD, USA) following with the manufacturer's instructions. The resulting RNA was used for the generation of labeled cDNA based on the protocol described earlier [44]. cDNA was probed against the Mouse GE 44K v2 Microarray G2519F-026655 (Agilent Technologies, Santa Clara, CA, USA) according to standard procedures, and the signals were read by the Agilent scanner and analyzed with Genespring software.

### Real time- polymerase chain reaction (RT-PCR)

Total RNA was isolated using the RNeasy Mini Kit (Qiagen) and 2 μg of the resulting RNA was used to synthesize cDNA using the SuperScript® III First-Strand Synthesis Systems (Invitrogen) as described by the manufacturer's instruction manual. RT-PCR was carried out with the ViiA™ 7 Real-Time PCR System (Applied Biosystems, Foster City, CA, USA). Sense and anti-sense primers specific for the genes of interest are listed in Suppl. Table 2. The transcript level of ribosomal protein L32 (RPL32) was used as an internal control. Note that, standard curve was used to quantify the CCK transcript level in human prostate cancer cell lines and transcript level was normalised as number of copies.

### Liquid chromatography–Mass spectrometry (LC-MS/MS)

Concentrated conditioned media (collected from fresh adipocyte or adipocyte co-cultured with TRAMP-C1 prostasphere) containing 19 ug of protein as quantitated by Pierce™ BCA Protein Assay Kit was separated on a SDS-PAGE to 8 mm and visualised by colloidal coomassie. Protein gel slices (1 mm) were excised using a clean gel cutter (The Gel Company San Francisco, CA, USA) and were transferred to 96-well U bottom plate containing a solution of 50% acetonitrile: 25mM NH4HCO3 for destaining. Samples were reduced with 20mM DTT followed by alkylation with 50mM IAA. The samples were equilibrated to pH-8 with 50 mM NH4HCO3 and subsequently dehydrated with 100% acetonitrile. Proteins were extracted with 60% acetonitrile, 1% formic acid, dried in a speed-vac and resuspended in 10 μL of 5% v/v formic acid for LC-MS/MS.

Peptides were analyzed using a 1200 Series nano HPLC and Chip-Cube Q-TOF 6510 (Agilent Technologies). HPLC loading pump was set to 3% B, flow rate of 4uL/min while analytical pump was set to 10%B and flow rate of 0.3uL/min. Mass spectrometry data were analysed using Spectrum Mill (Agilent, B.04.00.127) search engine. Extracted data were searched against Swiss-Prot Release 2012-01-25 database containing 534,242 sequence entries, with mouse species, fixed cysteine carbamidomethylation and variable methionine oxidation selected. Precursor and product mass tolerance was set to +/− 20ppm and ±=/− 50ppm respectively. Protein identification cut-offs were set to protein score > 11, peptide score >10 and scored peak intensity > 60% [45].

### ELISA

To quantify CCK and CTSB secretion, conditioned medium was collected and analysed using either the CCK Enzyme Immunoassay (EIA) Kit (Sigma-Aldrich) or the CTSB Mouse ELISA Kit (Abcam, Cambridge, MA, USA) following with manufacturer's instructions. Signals were detected using a LUMIstar OPTIMA Luminescence Microplate Reader. All results were then normalised as fold changes relative to the control.

## SUPPLEMENTARY MATERIAL AND METHODS FIGURES AND TABLES



## Abbreviations

Adi: Adipocytes
CSC: Cancer stem cells
CTSB: Cathepsin B
YM022: CCKBR inhibitor
CCK: Cholecystokinin
C-Adi: Co-cultured adipocytes
CM: Conditioned medium
CA-074ME: CTSB inhibitor
DMEM: Dulbecco's modified Eagle's medium
ELISA: Enzyme-linked immunosorbent assay
EGF: Epidermal growth factor
FABP4: Fatty acids binding protein 4
FBS: Fetal bovine serum
FGF: Fibroblast growth factor
FACS: Fluorescence-activated cell sorting
LC-MS/MS: Liquid chromatography–Mass spectrometry
MEM: Minimum Essential Medium
PMSF: Phenylmethylsulfonyl fluoride
PBS: Phosphate-buffered saline
PE: Phycoerythrin
RT-PCR: Real time- polymerase chain reaction
P/S: Penicillin-streptomycin
RPL32: Ribosomal protein L32
TBST-T: Tris Buffered Saline with Tween® 20
