# Supplementary material for: Adipocytes promote prostate cancer stem cell self-renewal through amplification of the cholecystokinin autocrine loop
Source: Oncotarget. 2015 Dec 17;7(4):4939–48. doi: 10.18632/oncotarget.6643 (PMC4826255; doi:10.18632/oncotarget.6643)
Supplement: Supplementary file 1 [file oncotarget-07-4939-s001.pdf]

SUPPLEMENTARY MATERIAL AND METHOD

CELL LINES AND CULTURE CONDITIONS

Mouse prostate cancer cell line TRAMP-C1 was obtained from ATCC (Rockville, MD, USA) and was maintained in RPMI 1640 medium (Invitrogen, Carlsbad, CA, USA) supplemented with 10% fetal bovine serum (FBS, Invitrogen) and 1% (wt/vol) penicillin-streptomycin (P/S, Invitrogen); whereas TC1-T5 was established as described in a previous study[1]. RM1 and RM1-BM were generous gifts from Dr Carl Power (University of New South Wales) and these cells were maintained in Dulbecco's modified Eagle's medium (Invitrogen) (DMEM containing 10% FBS, 1% P/S). Mouse pre-adipocyte (3T3-L1) and bone marrow stromal cell lines (OP9) were obtained from ATCC and were maintained in DMEM medium containing 10% FBS and 1% P/S and Minimum Essential Medium (MEM) α (Invitrogen) containing 20% FBS, 1% P/S respectively. Human prostate cancer cell line LNCaP was obtained from ATCC and was maintained in RPMI 1640 medium supplemented with 5% FBS and 1% P/S. C42B was kindly provided by Prof Leland Chung (Cedars-Sinai Medical Center) and was maintained in T-Medium (Invitrogen) supplemented with 5% FBS and 2% P/S. All cell lines were kept at 37°C in a 5% CO_2 environment.

ANTIBODIES AND REAGENTS

Human recombinant CCK protein was purchased from Tocris Biosciences (Bristol, United Kingdom). Human and mouse recombinant CTSB were purchased from R&D Systems (Minneapolis, MN, USA). CCKBR inhibitor (YM022) was purchased from Tocris Biosciences and CTSB inhibitor (CA-074ME) was purchased from Santa Cruz Biotechnology, Dallas, TX, USA. Both inhibitors were dissolved in DMSO.

The antibodies against Notch1, CD49f (Cell Signalling Technology, Danvers, MA, USA), Sca-1 (R&D Systems), Gama-tubulin (Sigma-Aldrich, St. Louis, MO, USA), Nanog, CCKBR and actin (Santa Cruz Biotechnology) were used in this study. Phycoerythrin (PE) conjugated Sca-1 antibody, PE Rat IgG2a, κ Isotype Control, Alexa Fluor® 647 Rat Anti-Human CD49f and Alexa Fluor® 647 Rat IgG2a κ Isotype Control were purchased from BD Biosciences (San Jose, CA, USA).

ADIPOCYTE DIFFERENTIATION

To obtain fully differentiated adipocytes, 3T3-L1 and OP9 cells were seeded into a 6-well plate at a confluency of 80%. Differentiation was induced with StemPro® Adipogenesis Differentiation Kit (Invitrogen) following manufacturer's instructions. Adipocyte differentiation medium was changed every 3 days, and the fully differentiated adipocytes were collected 9 days after the induction.

REFERENCES

1. Jeet V, Ow K, Doherty E, Curley B, Russell PJ, Khatri A. B roadening of transgenic adenocarcinoma of the mouse prostate (TRAMP) model to represent late stage androgen depletion independent cancer. Prostate. 2008; 68(5):548–562.

SUPPLEMENTARY FIGURES AND TABLES

Supplementary Table S1: Top-Ten list of the most upregulated genes in TRAMP-C1 cells after co-culturing with adipocytes

Primary Accession	Gene Symbol	Description	Fold Chage increased
**NM_031161**	**Cck**	**Mus musculus cholecystokinin (Cck), mRNA**	**1604**
NM_007817	Cyp2f2	Mus musculus cytochrome P450, family 2, subfamily f, polypeptide 2 (Cyp2f2), mRNA	1551
NM_010278	Gfi1	Mus musculus growth factor independent 1 (Gfi1), mRNA	1304
NM_007697	Chl1	Mus musculus cell adhesion molecule with homology to L1CAM (Chl1), mRNA	907
NM_009463	Ucp1	Mus musculus uncoupling protein 1 (mitochondrial, proton carrier) (Ucp1), nuclear gene encoding mitochondrial protein, mRNA	803
NM_011470	Sprr2d	Mus musculus small proline-rich protein 2D (Sprr2d), mRNA	689
NM_010819	Clec4d	Mus musculus C-type lectin domain family 4, member d (Clec4d), transcript variant 1, mRNA	610
NM_008077	Gad1	Mus musculus glutamic acid decarboxylase 1 (Gad1), mRNA	566
NM_011474	Sprr2h	Mus musculus small proline-rich protein 2H (Sprr2h), mRNA	458
NM_019549	Plek	Mus musculus pleckstrin (Plek), mRNA	445

Supplementary Table S2: List of the primers used in this study

Primers	Sequences
HCCKF	GGTACTCATACTCCTCGGCA
HCCKR	TGGCAAGATACATCCAGCAG
MALDH1A1	CAGCTAGCAGGTACTTCTG
MALDH1A1R	CCGATTACTGCAATCTTCATGG
MANG-1F	TGCCTACACTTTCATTCTTCC
MANG-1R	GACTGGTTCCTATCTCAAGCA
MCCKF	TTTAAGAGCAGTCACCCTCC
MCCKR	CTAGGACTGCCATCACCACG
MDKK2F	AAACTCAACTCCATCAAGTCCT
MDKK2R	CTTCACATTCCTTATCACTGCT
MGFI1F	ATCAAATGCAGCAAGGTCTTCTC
MGFI1	TCCGAGTGAATGAGCAGATGTG
MITGA2F	CCCAGAGCACTTTAGATTCCC
MITGA2R	GTGAACCAACCAGTAGCCAG
MITGA6F	TACAGCCTTCAACCTGGACAC
MITGA6R	CATCCACTGGTCTTCCTTGC
MPTHLHF	GGTATTCCTGCTCAGCTACTC
MPTHLHR	GTATCTGCCCTCATCGTCT
MSTC1F	AAAGCCACAACTTAGCGG
MSTC1R	ACAAATGTCGTACATCCCATCTG

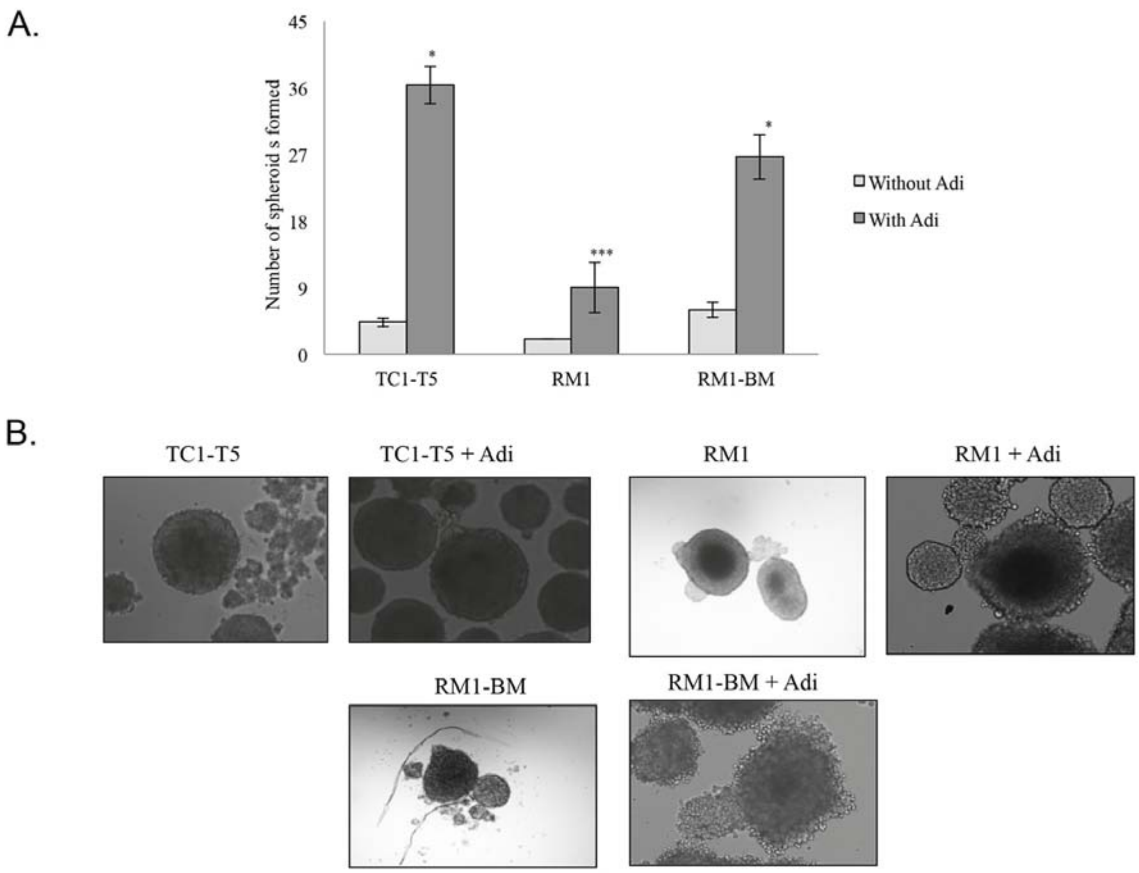

Supplementary Figure S1: Effect of adipocytes on self-renewal of mouse prostate cancer cell lines. A&B. Prostasphere formation assay was performed with mouse prostate cancer cell lines TC1-T5, RM1 and RM1BM in the presence or absence of 3T3-L1-derived adipocytes. Each experiment was repeated at least three times, and the results are presented as the mean ± SD. (p values: * < 0.05, *** < 0.0005).

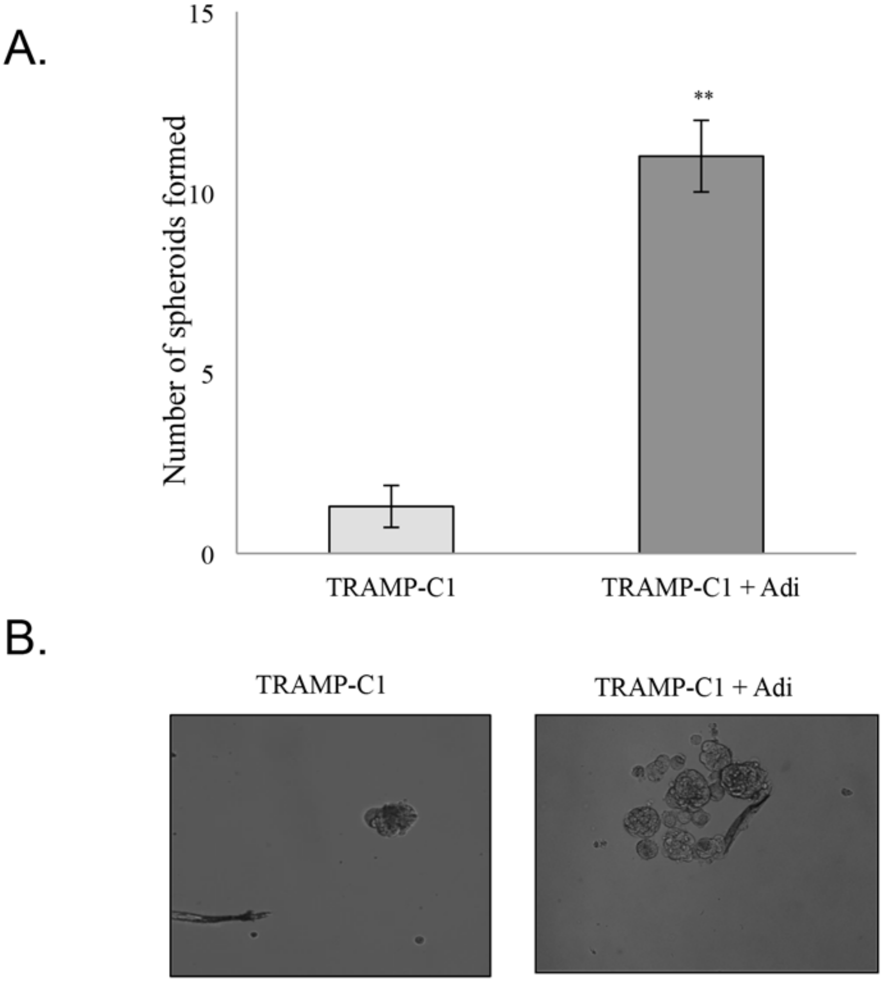

Supplementary Figure S2: Bone marrow-derived adipocytes promote prostate CSC self-renewal. A&B. TRAMP-C1 cells were seeded in ultra-low attachment plate in the presence or absence of OP9-derived adipocytes. After 7 days, prostaspheres formed were counted and imaged under the microscope. The results are presented as the mean ± SD from triplicate experiments. (p values: ** < 0.005).

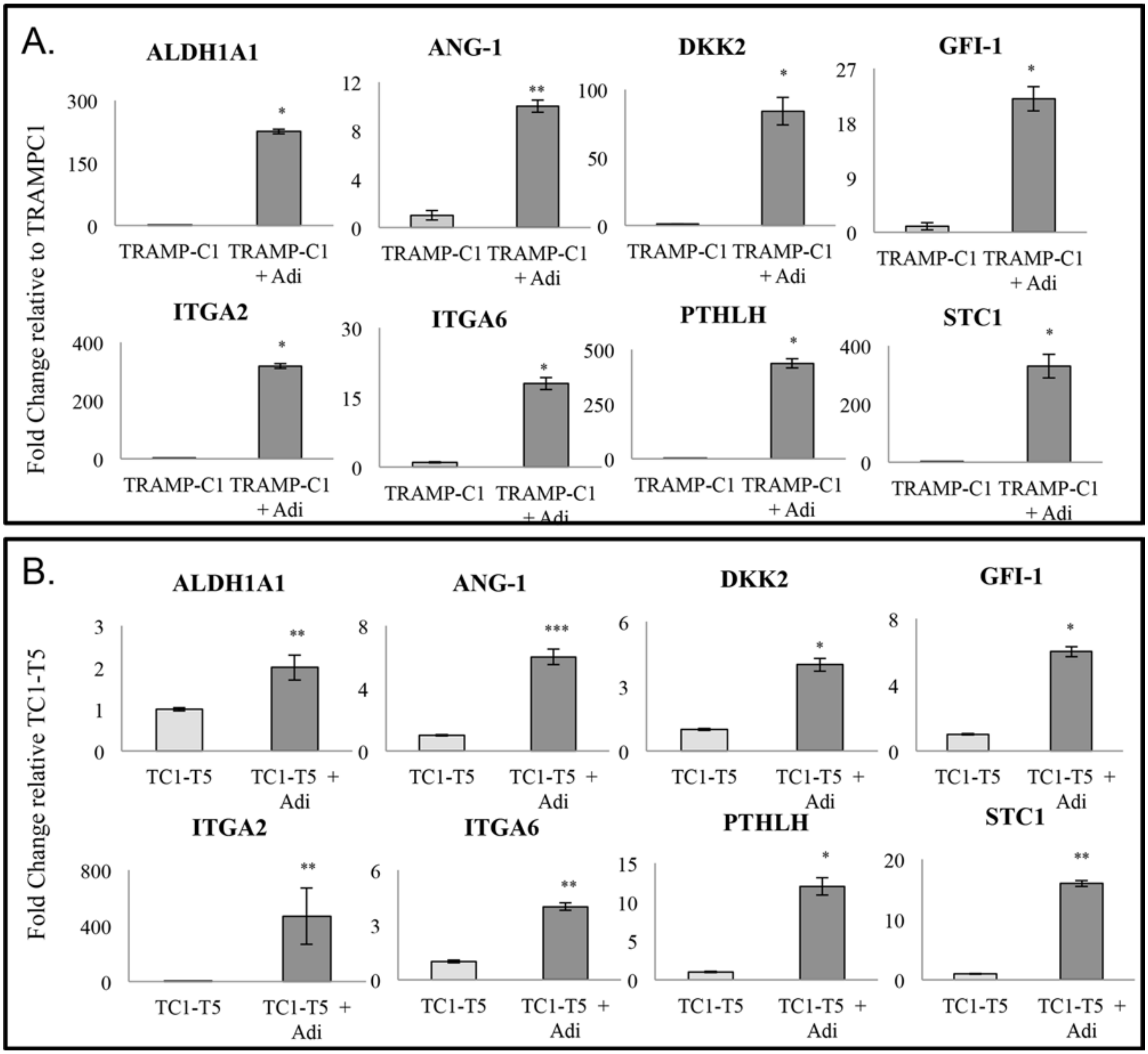

Supplementary Figure S3: CSC markers are upregulated in mouse prostate cancer cells that are co-cultured with adipocytes. RT-PCR analysis of CSC markers (ALDH1A1, ANG-1, DKK2, GFI-1, ITGA2, ITGA6, PTHLH and STC1) mRNA level in **A.** TRAMP-C1 and **B.** TC1-T5 cells that grown alone or co-cultured with adipocytes. The results are presented as the mean ± SD from triplicate experiments. (p values: * < 0.05, ** < 0.005, *** < 0.0005).

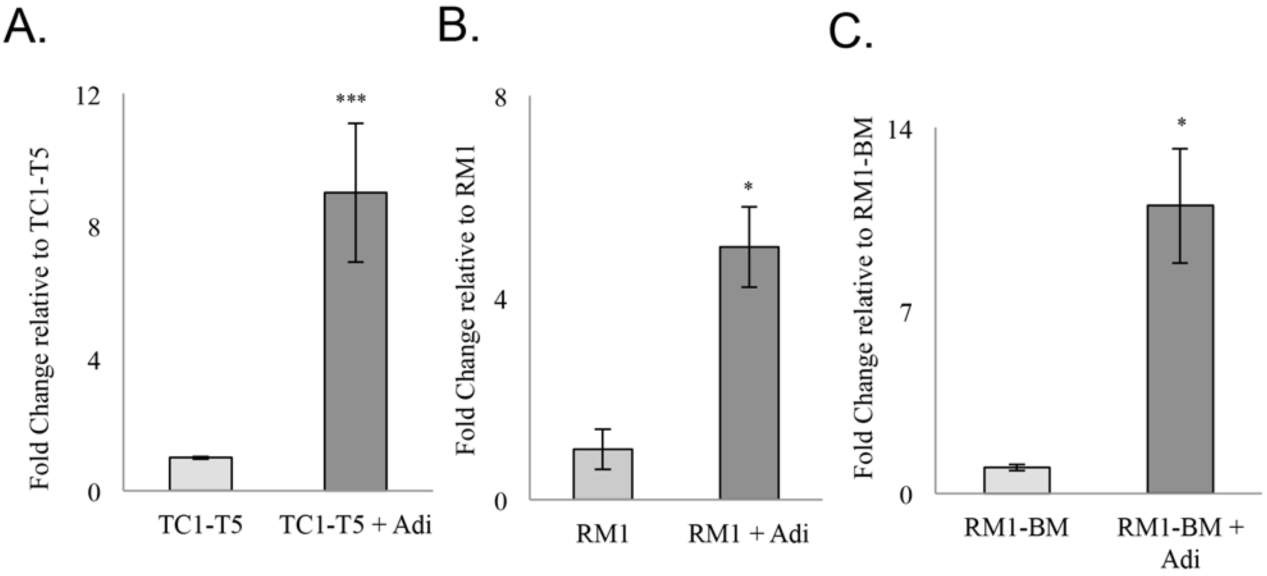

Supplementary Figure S4: CCK mRNA level is upregulated in mouse prostate cancer cells that are co-cultured with adipocytes. RT-PCR analysis of CCK mRNA level in **A.** TC1-T5, **B.** RM1 and **C.** RM1-BM cells that grown alone or co-cultured with adipocytes. The results are presented as the mean ± SD from triplicate experiments. (p values: ** < 0.005).

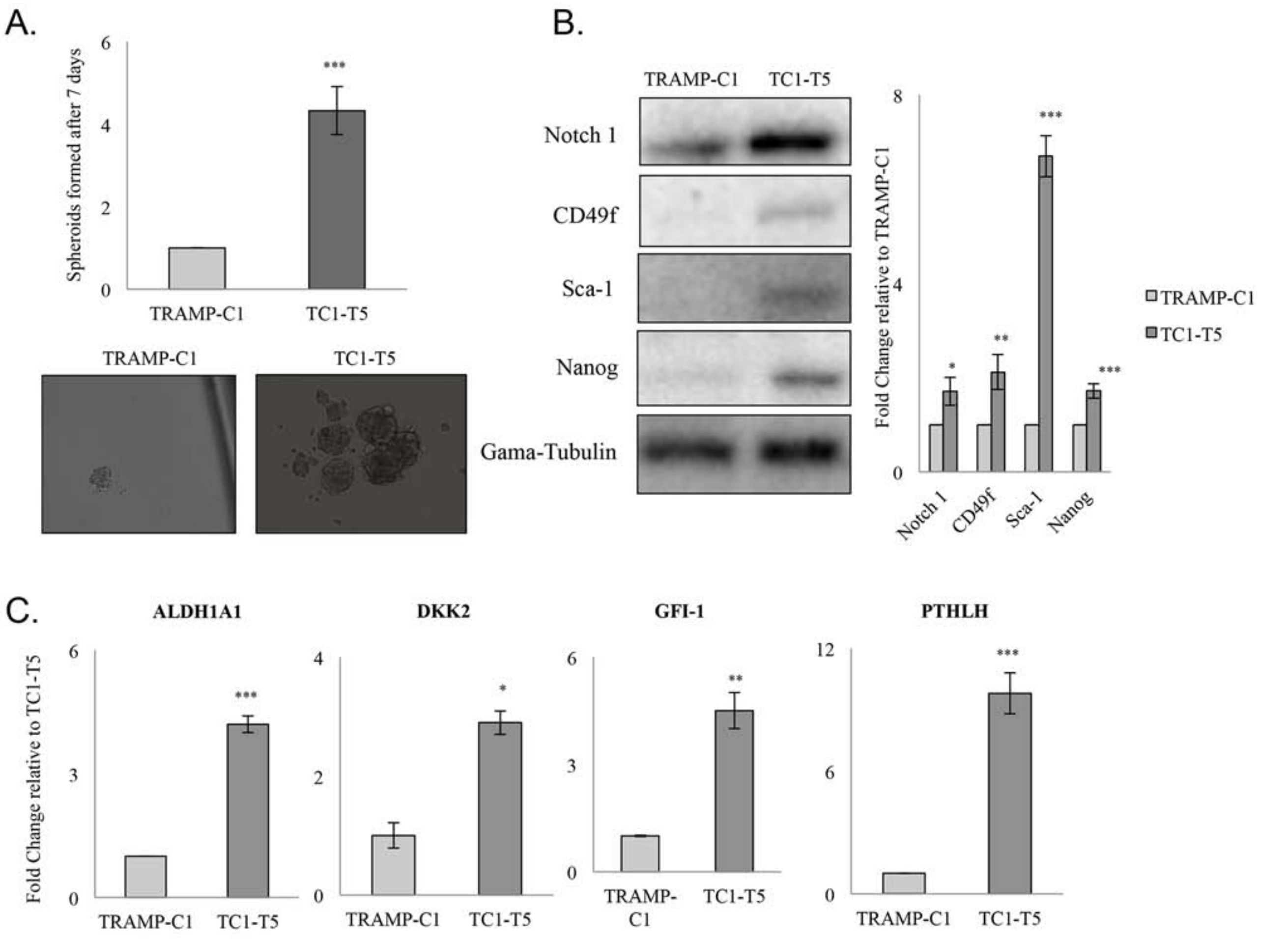

Supplementary Figure S5: Enrichment of CSCs in the bone metastatic cell lines TC1-T5. A. Self-renewal ability of TRAMP-C1 and its bone metastatic derivative TC1-T5 were examined by prostasphere formation assay. Prostaspheres formed at day 7 were counted and imaged under the microscope. **B&C.** Western blotting and flow cytometry were performed to examine the level of CSC markers expressed in the two cell lines. **D.** mRNA level of stem cell transcription factors (ALDH1A1, DKK2, GFI-1, HHIP and PTHLH) were analyzed with RT-PCR. Each experiment was repeated at least three times, and the results are presented as the mean ± SD. (p values: * < 0.05, ** < 0.005, *** < 0.0005).
